# Clinical analysis of human umbilical cord mesenchymal stem cell allotransplantation in patients with premature ovarian insufficiency

**DOI:** 10.1111/cpr.12938

**Published:** 2020-10-29

**Authors:** Long Yan, Yixuan Wu, Li Li, Jun Wu, Feiyan Zhao, Zheng Gao, Wenjing Liu, Tianda Li, Yong Fan, Jie Hao, Jianqiao Liu, Hongmei Wang

**Affiliations:** ^1^ State Key Laboratory of Stem Cell and Reproductive Biology Institute of Zoology Chinese Academy of Sciences Beijing China; ^2^ University of Chinese Academy of Sciences Beijing China; ^3^ Department for Stem Cell and Regeneration Chinese Academy of Sciences Beijing China; ^4^ Department of Gynecology and Obstetrics Key Laboratory for Major Obstetric Diseases of Guangdong Province The Third Affiliated Hospital of Guangzhou Medical University Guangzhou China; ^5^ National Stem Cell Resource Center Chinese Academy of Sciences Beijing China; ^6^ Department of Human Reproductive Medicine Beijing Obstetrics and Gynecology Hospital Capital Medical University Beijing China

**Keywords:** antral follicle, dominant follicle, follow‐up, mature follicle, premature ovarian insufficiency, stem cell therapy, umbilical cord mesenchymal stem cell

## Abstract

**Objective:**

Premature ovarian insufficiency (POI) is a refractory disease that seriously affects female fertility. Growing body of evidence has indicated mesenchymal stem cells (MSCs) as promising resources in regenerative medicine. In this study, we treated POI patients with umbilical cord‐derived MSCs (UCMSCs) and then investigated the restoration of ovarian function and clinical outcomes through follow‐ups.

**Materials and methods:**

Sixty‐one patients diagnosed with POI participated in this study. UCMSCs were isolated and cultured according to GMP standards, and then transplanted to the patients’ ovary by orthotopic injection under the guidance of vaginal ultrasound. We monitored side effects, vital signs and changes in clinical and collected haematological and imaging parameters during the follow‐ups.

**Results:**

All patients showed normal clinical behaviour without serious side effects or complications relevant to the treatment. Transplantation of UCMSCs rescued the ovarian function of POI patients, as indicated by increased follicular development and improved egg collection. POI patients who experienced shorter amenorrhoea durations (<1 year) seemed to obtain mature follicles more easily after stem cell therapy, and patients with better ovarian conditions (pre‐operative antral follicles) were more likely to derive the better outcomes by UCMSC injection. Four successful clinical deliveries were obtained from POI patients after UCMSC transplantation, and all of these babies are developed normally.

**Conclusions:**

The clinical trial result sugggests a possible therapy for POI by UCMSC transplantation.

## Graphical abstract

82 participants diagnosed of POI were screened for this study, 20 of them were ineligible as failure to meet the inclusion criteria, 1 patient declined. Take together, 61 patients were recruited. They experienced preoperative examination, UCMSCs transplantation and follow‐ups.

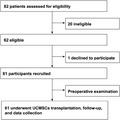

## 1 INTRODUCTION

Premature ovarian insufficiency (POI) is a disorder that causes infertility in women, affecting approximately 1% of the population.^1^ It is characterized by amenorrhoea, hypergonadotropism and hypoestrogenism before the age of 40.^2^ POI is highly heterogeneous and the aetiology remains poorly understood. Cause of POI including genetic, autoimmune, infectious and iatrogenic have been reported.^3^, ^4^, ^5^, ^6^, ^7^, ^8^ Patients usually have a significantly higher risk for bone loss^9^, ^10^, ^11^ and cardiovascular disease^12^, ^13^ due to long‐term oestrogen deficiency, and increased cardiovascular mortality raises the danger of premature death.^14^, ^15^, ^16^ Moreover, there are currently no effective treatments.

Hormone replacement therapy (HRT) is the most common treatment used to relieve oestrogen deficiency symptoms in the clinic,^17^, ^18^, ^19^, ^20^, ^21^, ^22^, ^23^ but it has limited effects on reproductive function. HRT is also controversial for long‐term usage, as several studies have reported that it confers increased risk of endometrial cancer,^24^, ^25^ ovarian cancer^26^ and breast cancer.^27^, ^28^, ^29^, ^30^ Ovarian tissue transplantation could restore endocrine function and fertility in patients with ovarian deficiency.^31^, ^32^ And autologous transplantation is primarily used in patients, with cryopreserving their ovarian tissues prior to chemo‐ or radiotherapy, resulting in an approximately 30% clinical pregnancy rate.^33^, ^34^, ^35^, ^36^ In vitro activation (IVA) of the dormant ovarian follicles in the POI ovary by AKT (also known as protein kinase B, PKB) stimulators enables POI patients to produce their own children,^37^, ^38^ though more experiments are needed to substantiate the effectiveness of the technique.^39^

Stem cell therapies seem to be promising for a wide spectrum of conditions, and they are expected to bring substantial benefit for patients suffering extensive diseases and injuries.^40^ To date, there have been more than 5600 clinical investigations registered using stem cell products (‘ClinicalTrials.gov’). MSCs are heterogeneous postnatal multipotent cells with the capacity of self‐renewal,^41^ and a variety of sources are used to isolate and manufacture MSC populations for clinical trials.^42^ Previous studies have shown that MSC transplantation can restore ovarian function and improve fertility in rodent models,^43^, ^44^, ^45^ 15 trials of clinical applications for POI and ovarian damage have been registered (‘ClinicalTrials.gov’), and it is very encouraging that 2 studies have reported successful delivery.^46^, ^47^ Nevertheless, the effectiveness of MSC treatment for POI needs further clinical validation. Moreover, more research is needed to identify the subgroup of patients who are most likely to benefit from this therapy.

In the present study, we transplanted GMP (good manufacturing practices) grade UCMSCs to the ovaries of POI patients by in situ injection. During the long‐term follow‐up, we found that UCMSCs were efficient in renovating the ovarian function and promoting oocyte maturation. After in vitro fertilization (IVF) and embryo transfer, 4 patients successfully conceived after transplantation, and the babies all developed normally. Most importantly, the results indicated that transplantation of UCMSCs rescued the ovarian function of POI patients by increasing follicular development and improving egg collection to some degree. Furthermore, patients who experienced shorter amenorrhoea time seemed more likely to benefit from the treatment, and the basic ovarian condition (such as pre‐operative antral follicle) was another important factor for the outcomes of stem cell therapy. In light of these findings, UCMSC transplantation may represent a promising approach for POI treatment in the future.

## 2 MATERIALS AND METHODS

### 2.1 Ethics

The study was approved prior to patient recruitment by the Ethics Committee of the Third Affiliated Hospital of Guangzhou Medical University and written informed consent was provided by all individual participants included in the clinical study. All experimental procedures were conducted in accordance with the guidelines of the Ethics Committee of the Third Affiliated Hospital of Guangzhou Medical University (No. 3, 2016) and complied with the Declaration of Helsinki and the standard ethical requirements of the local administrative department. The study was registered at ClinicalTrials.gov (NCT03033277).

### 2.2 Clinical study design

This non‐randomized clinical trial was conducted at the Third Affiliated Hospital of Guangzhou Medical University to assess the clinical outcomes of women with POI who were treated with intraovarian injections of UCMSCs. All patients received the standard hormone replacement regimen of estradiol (Femoston, Solvay pharmaceuticals BV, 2 mg/d) throughout the UCMSC treatment. All the individuals included in the study received intraovarian injections of UCMSCs in the first round of injection (n = 61); some continued to receive second intraovarian injections of UCMSCs (n = 50); and finally, some patients received third intraovarian injections of UCMSCs (n = 30). MSC injections contained 0.5 × 10^7^ UCMSCs in 100 µL of saline with 5% AB plasma for unilateral ovary and 1 × 10^7^ UCMSCs for bilateral ovaries. All groups were followed up for 6 months after injection of UCMSCs.

### 2.3 Patients

The diagnosis of POI was based on the following criteria from the ESHRE Guideline: (a) Oligo/amenorrhoea for at least 4 months; (b) an elevated FSH level >25 IU/L on two occasions >4 weeks apart. The population was recruited between July 2016 and December 2017 at the Third Affiliated Hospital of Guangzhou Medical University according to the following inclusion criteria: (a) ≤35 years old, with the intention of pregnancy; (b) previously regular menstrual cycle; (c) oligo/amenorrhoea for at least 4 months with an elevated FSH level >25 IU/L on two occasions >4 weeks apart; (d) married, with a husband that could produce spermatozoa. Patients were excluded according to the following criteria: (a) primary amenorrhoea; (b) history of severe drug allergy or autoimmune disease; (c) severe systemic disorders such as diseases of the cardiovascular system, liver, kidney and hematopoietic systems; (d) family history of severe genetic disease; (e) benign or malignant ovarian tumours; (f) anatomic abnormalities of the reproductive system that had a negative impact on pregnancy; (g) presence of contraindications to pregnancy; (h) infectious diseases such as positive antibodies against HIV or syphilis and viral hepatitis with abnormal liver function; (i) abnormal thyroid function; (j) history of malignancy; (k) abnormal karyotype; (l) current pregnancy or lactation; (m) alcohol or other drug abuse in the past 6 months; and (n) participation in other clinical trials within the previous 3 months.

### 2.4 Isolation and culture of UCMSCS

Healthy full‐term human placental samples were collected according to the policy of the Ethics Committee of the 306th Hospital of the Chinese People's Liberation Army, Beijing, China. Written informed consent was obtained from all donors before this study. The collected placenta was stored sterilely on ice and processed by the explant method within 4 hours after delivery. All the samples were used in accordance with standard experimental protocols approved by the Ethical Committee of Institute of Zoology, Chinese Academy of Sciences.

Briefly, the newborn umbilical cords were cut into small sections, and the veins and arteries were clearly removed. Then, the umbilical cords were cut to ~1 mm in size. The pieces of umbilical cords were directly transferred into 100‐mm plates in α‐MEM supplemented with 5% KOSR, 5 ng/mL bFGF and 1× NEAA. The plates were kept at 37°C in a 5% CO2 humidified atmosphere. After reaching approximately 80% confluence, UCMSCs were passaged. UCMSCs at passage 5 were utilized for all further experiments.

### 2.5 Identification of UCMSCs according to GMP standards

The isolated UCMSCs were performed according to the MSCs criteria proposed by the International Society for Cellular Therapies (ISCT). UCMSCs expressed MSC‐specific markers, and flow cytometry data demonstrated that UCMSCs highly expressed typical MSC markers (CD73, CD90 and CD105), showed low expression of endothelial and hematopoietic markers (CD34, CD45, CD19 and CD14) and were negative for the MHC class II molecule HLA‐DR. UCMSCs displayed the potential for trilineage differentiation into mesenchymal tissues, such as adipocytes, chondroblasts and osteoblasts.

### 2.6 Surgical and transplantation procedure

For pre‐operative preparation, the patient began a liquid diet (without milk, soyabean milk or other foods that would produce gas) the night before the operation. The UCMSC materials for this trial were transported from Beijing Stem Cells Bank to The Third Affiliated Hospital of Guangzhou Medical University. The viability of the thawed cells were tested, and the density was adjusted to 0.5 × 10^7^/100 µL immediately after arrival at the hospital.

Patients emptied their bladders and bowels before surgery. The vulva, vagina and cervix were routinely disinfected with iodophor, and sterile drapes were whisked onto the patients’ chests and pelves. One surgeon and one ultrasound physician jointly determined the location and the puncture route of the ovary. A 21G needle (321300, Kitazato BioPharma Co., Ltd.) was employed for puncture, with the other end connected with the hose of a 100‐µL syringe that was previously invented and patented and was utilized in this operation. After emptying the air in the puncture system by stem cell suspension, the surgeon used the needle for puncture under transvaginal ultrasonographic (TVUS) guidance. Each ovary was injected at three points, with 35 µL of UCMSCs per point. Each patient in different groups received up to three transplantations at different intervals in line with the study protocol.

### 2.7 Ultrasound evaluation of ovaries

Repeated transvaginal ultrasonographic examinations were performed by an expert to monitor ovarian follicular growth using a GE ultrasound system and a 6.5 MHz probe (Voluson P8, GE). We also recorded the mean diameter of the ovaries, which was calculated as follows: D=[Lmaximallength)+W(maximalwidth)]/2, and the mean diameter of follicles was defined as follows: D=[Lmaximallength)+W(maximalwidth)]/2.

### 2.8 Follow‐up

Clinical outcomes and trial assessments were evaluated in the following 6 months by another surgeon in the study, who was not involved in the treatment procedures and was blinded to the patient allocation. Follicular development was examined by transvaginal ultrasound on the 3rd and 14th days of the menstrual cycle. If follicles were found in the ovaries, HMG (Lebaode, Livzon Pharmaceutical Factory) 300 IU/d was administered for ovarian stimulation. Serum AMH, FSH, E2, LH, liver function (ALT, AST), renal function (Scr, BUN) and routine blood test were examined on the 3rd day of menstruation each month. Examinations included ultrasound of the ovaries, uterus and breast, ECG, routine blood tests, routine coagulation tests, hepatitis B and C, AIDS, syphilis, tumour markers, liver and kidney function were performed before enrollment and 6 months after stem cell therapy.

### 2.9 Outcomes

The primary outcome of the trial was the number of matured follicle (MFC) per month. The secondary outcomes of the trial included the number of dominant follicles (DFC) and antral follicle counts (AFC) per month; the size of ovaries; anti‐*Müllerian* hormone (AMH), follicle‐stimulating hormone (FSH), luteinizing hormone (LH) and estradiol (E_2_) levels in serum; the number of pregnancies and live births; the number of oocytes retrieved, matured oocytes, high‐quality embryos, rates of fertilization, clinical pregnancy, miscarriage and live birth for IVF/ICSI cycles; and the number of adverse events, such as fever, rash, pelvic bleeding, headache, infectious diseases, neoplasms, abnormal liver function and abnormal renal function.

### 2.10 Hormonal assays

Blood samples were obtained on the 3rd day of menstruation each month. Serum samples were stored at −80°C until assayed. Hormone levels were measured from stored samples. Radioimmunoassay (RIA, Diagnostic Products Corporation) was used to quantify serum levels of AMH, FSH, LH and E2. Serum E2 was measured by RIA with a sensitivity limit of 17 pg/mL and interassay coefficients of variation = <7%. The sensitivity and interassay coefficients of variation of serum FSH and LH were 0.05 IU/L and <10%, while the two parameters of serum AMH were 0.06 ng/mL and <15%, respectively.

### 2.11 Statistical analysis

All statistical analyses were performed using SPSS (Statistical Package for the Social Sciences) version 22.0 (IBM). Quantitative variables with homogenous variance are expressed as $\bar{X}\pm \text{SD}$, and the means were compared by Student's *t* test or ANOVA. Quantitative variables with heterogeneous variance are expressed as medians (1st quartiles, 3rd quartiles), and the medians were compared by the Mann‐Whitney *U* test or Kruskal‐Wallis test. Repeated measures of ANOVA was used to analyse the differences in continuous variables at multiple time points among the groups. A logistic regression model was performed to identify potential predictors for the failure to obtain mature follicles. For all tests, *P* < .05 was considered significant.

## 3 RESULTS

### 3.1 Patient characteristics

**FIGURE 1:**
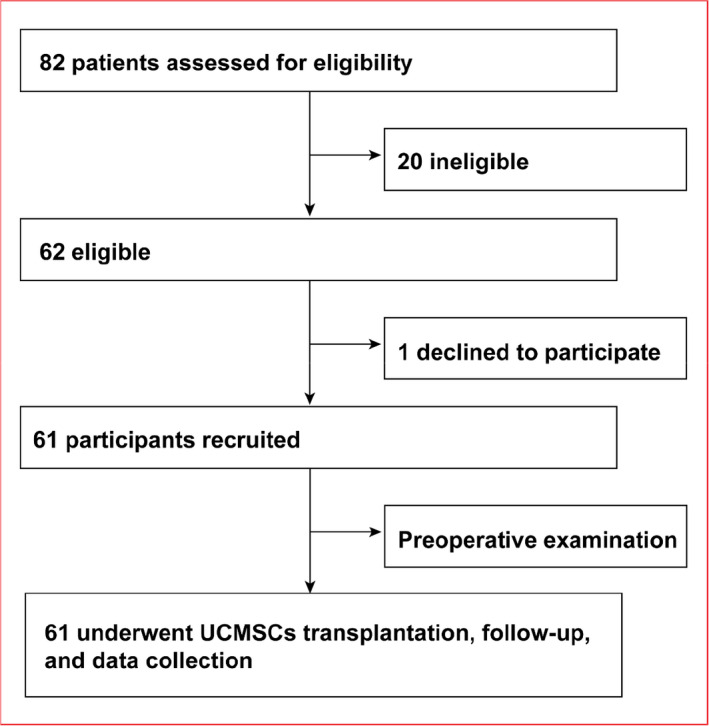
Participant flow chart

In this study, a total of eighty‐two patients were enrolled. Twenty were not eligible due to pathologic conditions such as endometriosis, endocrine disorders or other gynaecological diseases. Sixty‐two women were eligible, but one patient declined to participate in the trial. Finally, 61 patients were recruited and completed pre‐operative examinations to ensure basic conditions (Figure 1). The demographic characteristics of the participants are shown in Table 1. Overall, before the intraovarian injections of UCMSCs, patients enrolled in the study had similar ages, years of amenorrhoea, BMI (body mass index), history of gestation, hormone levels and ovarian follicles in order to ensure comparability in the following treatment. Two UCMSC strains used in the trial were isolated from healthy full‐term human placental samples and qualified to be clinically used by the National Institutes for Food and Drug Control.

**TABLE 1:** Participants’ characteristics and cell line usage

Baseline Characteristics	Total (n = 61)
Age (y)	31 (29, 34)
Amenorrhoea duration (y)	3 (1.5, 6)
BMI (kg/m^2^)	21.5 (20, 24.3)
Pregnancy history	0 (0, 1)
Delivery history	0 (0, 0)
E2 (pmol/L)	40 (37, 102)
LH (IU)	25.7 (19.22, 33.7)
FSH (IU)	53.12 (42.42, 63.72)
AMH (ng/mL)	
<0.06	45 (73.8%)
0.06‐0.54	16 (26.2%)
Ovary diameter (mm)^a^, ^b^	13 (11.8, 16.5)
Ovary diameter (mm)^a^, ^c^	13.5 (11.5, 15)
AFC^b^	
0	49 (80.3%)
1‐3	12 (19.7%)
AFC^c^	
0	46 (75.4%)
1‐3	15 (24.6%)
AFC (total)	
0	42 (68.9%)
1‐5	19 (31.1%)
Cell line	
1	21 (34.4%)
2	40 (65.6%)

Note

AFC was categorical variable, which was divided into 0 and more than or equal to 1. Total = b + c.

Abbreviations: AFC, antral follicle count; BMI, body mass index.

^a^ Mean diameter.

^b^ Left ovary.

^c^ Right ovary.

### 3.2 Primary outcome

UCMSCs were transplanted to the patients by ultrasound‐guided transvaginal injection (Video S1), and the outcomes were defined as the number of mature oocytes per month after the stem cell therapy (Table 2). All patients received the first round of operation; however, some of them failed to receive the second and third rounds of transplantation, so several cases were lost to follow‐up. The data of AMH, antral follicle (AFC), number of the dominant follicles (DFC) and mature follicles (MFC) were collected each month after the operation. Particularly, we could only acquire six patients’ outcomes five months after the first transplantation; therefore, we integrated the data of the fifth and sixth months into one item to be more visible. As a result, 31.1% (19/61), 11.8% (6/50) and 13.3% (4/30) of the patients obtained matured follicles after the first, second and third operations, respectively. There was an obvious trend for patients to gain improvements in follicular development, which included an ascending of AFC and increased numbers of DFC and MFC, together with elevated AMH levels despite the exclusion of lost follow‐ups. Fifty patients had a second transplantation after a rigorous clinical evaluation. Thirty patients participated the third round operation. Similarly, patients took part in the last two injections seemed to achieve better ovarian functions with regard to the indexes of relevant follicle counts. Additionally, three patients received a unilateral injection because of the invisibility of the opposing ovary through transvaginal ultrasonography (Table S1). Intriguingly, ovaries underwent UCMSC transplantation all derived antral follicles, while no AFCs were found in the ovaries without injection.

**TABLE 2:** Follicle development after stem cell therapy

	Follow‐up (mo)					
	1	2	3	4	5 and 6	1‐6
One transplant (n = 61)	61	56	29	21	6	61
AMH ≥ 0.06 (ng/mL)	7 (11.7%)	11 (21.6%)	4 (14.8%)	8 (42.1%)	1 (16.7%)	
AFC	14 (23%)	19 (33.9%)	12 (41.4%)	10 (47.6%)	3 (50%)	34 (55.7%)
DFC	2 (3.3%)	6 (10.9%)	2 (6.9%)	3 (14.3%)	1 (16.7%)	13 (21.3%)
MFC	6 (9.8%)	10 (17.9%)	3 (10.3%)	4 (19%)	2 (33.3%)	19 (31.1%)
Total	18 (29.5%)	27 (48.2%)	16 (55.2%)	13 (61.9%)	4 (66.7%)	41 (67.2%)
Two transplants (n = 50)	50	36	22	15	12	50
AMH ≥ 0.06 (ng/mL)	5 (10.4%)	5 (13.5%)	3 (17.6%)	2 (15.4%)	8 (66.7%)	
AFC	13 (26.0%)	5 (13.9%)	9 (40.9%)	6 (40.0%)	4 (33.3%)	21 (41.2%)
DFC	3 (6.0%)	2 (5.6%)	1 (4.5%)	2 (13.3%)	2 (16.7%)	5 (9.8%)
MFC	3 (6.0%)	1 (2.8%)	1 (4.5%)	3 (20.0%)	4 (33.3%)	6 (11.8%)
Total	13 (26.0%)	6 (16.7%)	9 (40.9%)	8 (53.3%)	6 (50%)	22 (43.1%)
Three transplants (n = 30)	30	26	26	20	19	30
AMH ≥ 0.06 (ng/mL)	3 (10.3%)	2 (7.7%)	1 (4.3%)	1 (5.6%)	1 (5.3%)	
AFC	4 (13.3%)	4 (15.4%)	4 (15.4%)	1 (5.0%)	6 (31.6%)	13 (43.3%)
DFC	1 (3.3%)	0 (0.0%)	0 (0.0%)	0 (0.0%)	0 (0.0%)	1 (3.3%)
MFC	2 (6.7%)	1 (3.8%)	0 (0.0%)	0 (0.0%)	1 (5.3%)	4 (13.3%)
Total	5 (16.7%)	5 (19.2%)	4 (15.4%)	1 (5%)	6 (31.6%)	15 (50.0%)

Note

Quantitative variables with heterogeneous variance were expressed as median (1st quartiles, 3rd quartiles), categorical variables were expressed as N (%). Total = AFC + DFC + MFC.

Abbreviations: AFC, antral follicle count; AMH, anti‐*Müllerian* hormone; DFC, dominant follicle count; MFC, mature follicle count.

UCMSCs isolated from different sources may have heterogeneity. In this study, twenty‐one patients received cells from line 1, while the other 40 patients from line 2 (Table S2). Overall, 7 (33.3%) and 12 (20.0%) patients were found MFC development in the 2 groups after different UCMSC transplantation, with no significant difference. Both groups had generally similar increase in the number of patients with MFC at each follow‐up posterior to the three operations. Among the three transplants, no statistical difference of MFC availability was found between the 2 cell lines. However, there was a significantly difference among the five follow‐ups (*P*_time_ = .007) after the second transplant, which showed obviously more MFC development in the 5th follow‐up than others.

### 3.3 Oocyte retrieval and pregnancy characteristics

**FIGURE 2:**
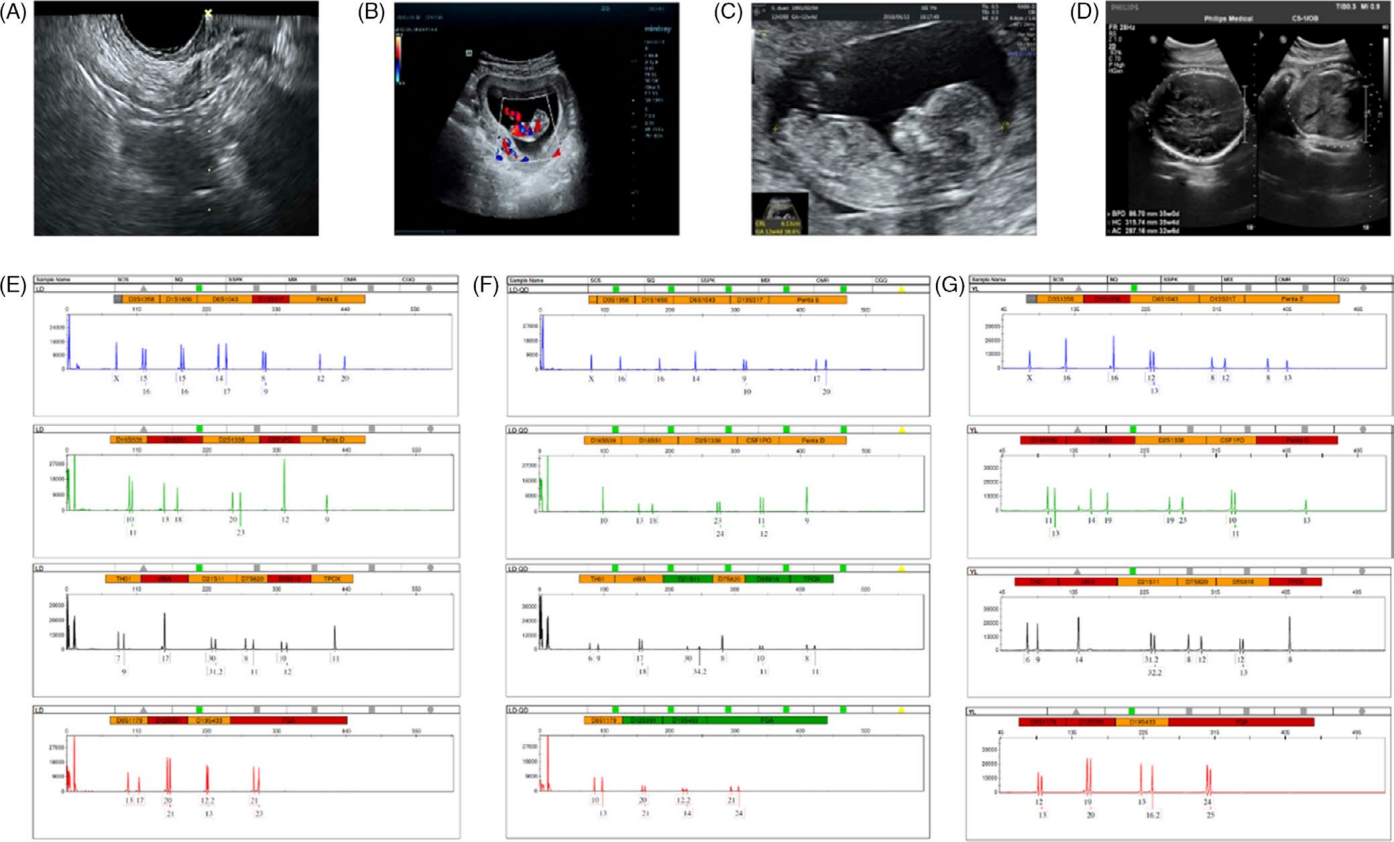
Pregnancy and neonatal outcomes. (A) Ultrasound scans showed puncture process during USMSCs transplantation; (B‐D) Ultrasonographic of a patient with pregnancy at gestational age of 8 wk (B), 13 wk (C) and 32 wk (D); (E‐F） Microsatellite loci analysis of maternal blood (E), foetus’ umbilical cord (F) and donor cells (UCMSCs) (G)

Basic characteristics of 15 patients who received oocyte retrieval were collected (Table S3), including retrieval cycles, embryos transplantation and numbers of pregnancies. Generally, four foetuses were delivered without birth defects, three embryos came from ICSI for the type of fertilization (Table S4), while the last one was naturally conceived. Ultrasonographic scanning of one case during the surgery revealed no stromal hyperplasia or other abnormal ovarian structures in the ovaries under transvaginal ultrasonographic (TVUS) guidance (Figure 2A). This woman was successfully pregnant after the in vitro fertilization, and ultrasound scans of basic prenatal procedures on the 8th week (Figure 2B), 13rd week (Figure 2C) and 32nd week (Figure 2D) showed normal foetal development. Microsatellite loci analysis revealed that the foetus (Figure 2F) was genetically related to the mother (Figure 2E) other than the donor UCMSC (Figure 2G).

### 3.4 Correlations between primary outcome and baseline characteristics

To confirm the relationship between the therapeutic efficacy and the baseline characteristics, we compared the basic clinical characteristics between 19 patients who gained mature follicles after UCMSC injecting and the other 42 patients who had no mature follicles developed. Patients with MFC have a median age of 30 and amenorrhoea duration of 1.3 years. The blood hormone level of E2 in these MFC patients was trended towards higher than no MFC group, with no difference of LH and FSH levels between the two groups. As a valid indicator of ovarian reserve, AMH level was also tested. In general, 36.8% of the MFC group had a AMH level ≥0.06 ng/mL, while the proportion was 21.4% in the none MFC group. The significant differences of amenorrhea duration and AFC before transplantation between these two groups indicated they were the two major factors affecting therapeutic efficacy (Table 3).

**TABLE 3:** Baseline characteristics of patients with and without MFC

Characteristics	MFC (n = 19)	No MFC (n = 42)	*P*
Age (y)	30 (29, 32)	32.5 (28.8, 34)	.112
Amenorrhoea duration (years)	1.3 (0.33, 3)	4 (2, 7.3)	.001**
BMI (kg/m^2^)	21.1 (19.7, 22.7)	21.8 (20.2, 24.5)	.383
Pregnancy history	0 (0, 0)	0 (0, 1)	.418
Delivery history	0 (0, 0)	0 (0, 0)	.535
E2 (pg/mL)	55 (37, 117)	38 (37, 85.87)	.256
LH (IU)	26.09 (17.55, 31.89)	25.46 (19.21, 35.13)	.647
FSH (IU)	57.8 (42.42, 62.8)	49.29 (41.31, 65.42)	.815
AMH (ng/mL)			
<0.06	12 (63.2%)	33 (78.6%)	.341
≥0.06	7 (36.8%)	9 (21.4%)	
Ovary diameter (mm)^a^, ^b^	13 (11.8, 16.8)	12.5 (11.3, 16.3)	.435
Ovary diameter (mm)^a^, ^c^	13 (11.5, 14.8)	13.5 (11.5, 15)	.739
Total AFC			
0	8 (42.1%)	34 (81.0%)	.002**
1‐3	11 (57.9%)	8 (19.0%)	
Cell line			
1	7 (36.8%)	14 (33.3%)	.789
2	12 (63.2%)	28 (66.7%)	

Note

Non‐normally distributed continuous variables were expressed as median (1st quartiles, 3rd quartiles), and statistical differences were calculated using Mann‐Whitney test or Kruskal‐Wills test; categorical variables were expressed as N (%), and statistical differences were calculated using χ2 or Fisher's exact test; total AFC was categorical variable, which was divided into 0 and more than or equal to 1.

Abbreviations: AFC, antral follicle count; AMH, anti‐*Müllerian* hormone; BMI, body mass index.

^a^ Mean diameter.

^b^ Left ovary.

^c^ Right ovary.

* *P* < .05.

** *P* < .01.

*** *P* < .001.

### 3.5 Exploration of factors affect the efficacy of stem cell therapy

Based on the findings aforementioned, we next analysed the effects of amenorrhoea time and pre‐operative AFC on the primary outcome. First, we divided the patients into three groups (less than 1 year, 1‐3 years and more than 3 years) according to the menopause period (Table 4). MFC acquisition of the three groups after the first operation were 70%, 41.2% and 14.7%, respectively (*P* = .002); MFC acquisitions after the second transplantation were 33.3%, 20% and 3.1% (*P* = .024), while the number after the third transplantation were 33.3%, 16.7% and 5.6% (*P* = .163). The results indicated that during the first two rounds of UCMSC transplantation, patients who had amenorrhoea over three years exhibited significantly lower frequency of MFC and DFC developing. Then, we compared the characteristics of 19 patients who had antral follicles before the surgery with those who did not (Table S5). Age, amenorrhoea duration, BMI, history of pregnancy and delivery, sex hormones levels, ovary diameters were taken into account. Amenorrhoea duration (2 (0.33, 3) vs 4 (2, 9), *P* = .001) and ovary diameters (left ovary: *P* = .001; right ovary: *P* < .001) were found significantly different between these two groups, suggesting these 2 baseline characteristics could be used as indicator for pre‐operative AFC.

**TABLE 4:** Amenorrhoea duration and therapeutic effect

Amenorrhoea duration (y)	<1 (n = 10)	1‐3 (n = 17)	≥3 (n = 34)	*P*
First transplant				
AFC	7 (70.0%)	9 (52.9%)	18 (52.9%)	.611
DFC	5 (50.0%)	6 (35.3%)	2 (5.9%)	.002**
MFC	7 (70.0%)	7 (41.2%)	5 (14.7%)	.002**
AFC and DFC and MFC	9 (90.0%)	11 (64.7%)	21 (61.8%)	.239
Second transplant				
AFC	5 (55.6%)	3 (30.0%)	13 (40.6%)	.556
DFC	3 (33.3%)	2 (20.0%)	0 (0.0%)	.003**
MFC	3 (33.3%)	2 (20.0%)	1 (3.1%)	.024*
AFC and DFC and MFC	5 (55.6%)	3 (30.0%)	14 (43.8%)	.562
Third transplant				
AFC	1 (16.7%)	2 (33.3%)	10 (55.6%)	.273
DFC	0 (0.0%)	0 (0.0%)	1 (5.6%)	1.000
MFC	2 (33.3%)	1 (16.7%)	1 (5.6%)	.163
AFC and DFC and MFC	3 (50.0%)	2 (33.3%)	10 (55.6%)	.875
Total				
AFC	8 (80.0%)	10 (58.8%)	24 (70.6%)	.491
DFC	6 (60.0%)	7 (41.2%)	3 (8.8%)	.001**
MFC	7 (70.0%)	7 (41.2%)	5 (14.7%)	.002**
AFC and DFC and MFC	9 (90.0%)	11 (64.7%)	25 (73.5%)	.343

Note

Categorical variables were expressed as N (%). Statistical differences were calculated using χ2 or Fisher's exact test. Total = follicle outcomes of all three transplants.

Abbreviations: AFC, antral follicle count; DFC, dominant follicle count; MFC, mature follicle count.

* *P* < .05.

** *P* < .01

*** *P* < .001.

Age is generally considered as a vital factor affects reproductive outcome in women. Therefore, we divided the patients into three groups according to female age (Table S6). There were no significant differences among the three groups regarding to the numbers of antral, dominant and matured follicles except the group consisting of women more than 35 years gained a superior AFC after the third transplantation. We also analysed the parameters of follicular development among different BMI groups. Nevertheless, no significant differences were presented among the three groups (Table S7).

In addition, we used an adjusted multivariable logistic regression model for failure of MFC availability to further validate the main effective indicators (Table 5). We first performed univariate logistic regression and found that amenorrhoea duration (*P* = .021) and pre‐operative AFC (*P* = .004) were potential confounders, which is consistent with the above findings. Next, the multivariate logistic regression model suggested total AFC be regarded as an independent potential factor affecting MFC development during this therapy (OR, 0.188, 95% CI, 0.057, 0.621, *P* = .006).

**TABLE 5:** Logistic regression analysis of risk factors affecting the main outcomes

Characteristics	Single factor analysis			Multi‐factor analysis		
	OR	95% CI	*P*	OR	95% CI	*P*
Age (years)	1.136	(0.951, 1.357)	.161			
Amenorrhoea duration (years)	1.298	(1.041, 1.619)	.021*			
BMI (kg/m^2^)	1.081	(0.912, 1.282)	.369			
Pregnancy history	1.780	(0.637, 4.972)	.271			
Delivery history	1.700	(0.318, 9.075)	.535			
E2 (pg/mL)	0.998	(0.994, 1.003)	.518			
LH (IU)	1.007	(0.963, 1.053)	.753			
FSH (IU)	1.001	(0.975, 1.029)	.916			
AMH (ng/mL)	0.467	(0.142, 1.534)	.210			
Ovary diameter (mm) ^a^, ^b^	0.929	(0.789, 1.095)	.382			
Ovary diameter (mm) ^a^, ^c^	0.976	(0.824, 1.156)	.782			
AFC (Total)	0.171	(0.052, 0.564)	.004**	0.188	(0.057, 0.621)	.006**
Cell line	0.857	(0.276, 2.658)	.789			
Number of transplants	0.776	(0.365, 1.65)	.510			

Note

Age, amenorrhoea duration, BMI, pregnancy history, delivery history, E2, LH, FSH and ovary diameter were non‐normally distributed continuous variables. AMH: total AFC and number of transplants were categorical variables.

Abbreviations: AFC, antral follicle count; AMH, anti‐*Müllerian* hormone; BMI, body mass index; CI, confidence interval; OR, odds ratio.

^a^ Mean diameter.

^b^ Left ovary.

^c^ Right ovary.

* *P* < .05.

** *P* < .01.

*** *P* < .001.

### 3.6 Adverse events

The safety of stem cell therapy is the most important concern during clinical application. In this trial, all patients showed no serious side effects or complications relevant to the treatment. However, five patients experienced mild sequelae which were transient and responsive to rest and simple therapeutic interventions (Table 6). In detail, one patient had vaginal bleeding 6 hours after the transplantation operation, and the bleeding stopped after compression with gauzes; one patient had slightly elevated white blood cell counts and increased percentage of neutrophils without fever and abdominal pain. She recovered after taking a 3‐day course of cefuroxime axetil tablets; one patient was found to have elevated levels of alanine aminotransferase (ALT) and glutamic oxaloacetic transaminase (GOT) the day after the operation, and she recovered 3 months after the therapy; one patient had vaginal itching 3 days after the operation, which resolved without treatment; another patient had candida vaginitis and recovered after 1 week of clotrimazole treatment.

**TABLE 6:** Adverse events

Adverse events	Quantity
Fever	0
Rash	0
Pelvic bleeding	0
Vaginal bleeding	1
Headache	0
Infectious diseases	1
Neoplasms	0
Abnormal liver function	1
Abnormal renal function	0
Vaginitis	2

## 4 DISCUSSION

In this study, we performed UCMSC transplantation in 61 POI patients and carried out follow‐ups to validate the safety and effectiveness of this treatment, and no patients showed obvious complications related to this therapy. As expected, different stages of follicles (AFC, DFC and MFC) were found to grow in the ovaries after stem cells injection. Besides, three patients received unilateral injection showed follicle development in the injection ovary but no developing follicle appeared in the other ovary without UCMSC transplantation, which further indicated effectiveness of UCMSC treatment. Furthermore, two patients resumed normal menstruation, one of them was found more than 10 antral follicles developed in each ovary during the recent follow‐up (data not shown), suggesting completely ovarian function restoration. All of the results indicate a promising application future of UCMSCs on clinical treatment of POI disease.

Although majority of the patients completed the trial benefiting from the stem cell treatment, our results also found some unsatisfactory efficacy on certain cases. For instance, patients who had amenorrhoea over three years seemed difficult to get obvious therapeutic effect (follicular development). Usually, this group of patients mainly endured more severe endocrine disorders and ovarian deficiency which probably make it difficult to restore the ovarian function only by UCMSCs in a short term. It is noteworthy that patients with advance aged derived more AFC than other groups after the third transplantation (Table S6), which might partly attribute to a better efficacy on primordial activation in older women, albeit a small sample size of this group. Besides, we performed a logistic regression to identify potential factors related to MFC acquisition. Amenorrhoea duration and ovarian AFC were considered as risk factors in single factor analysis, though only total AFC was still significant in multi‐factor analysis, which implied the presence of AFC before surgery could predict the difficulty of MFC availability after UCMSC transplantation. Conceivably, the patients with remaining AFC prior to operation may get more benefit from the UCMSCs therapy. Our results warrant a larger, randomized trial with long‐term follow‐up to be performed in the future.

Numerous preclinical studies have investigated the possible mechanism underlying the effects of MSC administration on ovarian dysfunction in various animal models. MSCs could secrete growth factors, including vascular endothelial growth factor (VEGF), fibroblast growth factor‐2 (FGF‐2), angiogenin, epidermal growth factor (EGF), insulin‐like growth factor‐1 (IGF‐1), hepatocyte growth factor (HGF), which played a central role in upregulating B‐cell lymphoma‐2 (Bcl‐2), reducing apoptosis in stromal cells or granulosa cells, facilitating neovascularization and inhibiting ovarian aging.^48^, ^49^, ^50^, ^51^ Excessive fibroblast proliferation and extracellular matrix (ECM) deposition form ovarian fibrosis, which is usually associated with POI progression. MSCs have demonstrated a certain anti‐fibrotic effect by regulating fibrosis‐related cytokines such as MMPs, tissue inhibitors of MMPs (TIMPs), transforming growth factor‐β1 (TGF‐β1) and endothelin‐1 (ET‐1).^52^, ^53^ Furthermore, anti‐inflammation and immunomodulatory effects may be the other mechanism by which MSCs restore ovarian function. MSC transplantation could upregulate anti‐inflammatory regulatory T (Treg) cells and inhibit pro‐inflammatory Th17 cells.^54^ Upregulation of prostaglandin E2 (PGE2) and expression of indolamine 2,3‐dioxygenase (IDO) may be implicated in MSC‐mediated immunosuppression by inhibiting macrophages or dendritic cells (DCs).^55^ Although the stem cell therapy for POI using MSCs has been extensively studied, the detailed molecular mechanism remains to be further elucidated.

In conclusion, our trial suggested that UCMSCs improves follicular growth especially in patients with a shorter amenorrhoea duration before transplantation. The therapy effectively rescued the ovarian function, with no severe adverse events. Despite some problems raised by the current research need to be further confirmed, such as the duration of stem cells efficacy, distinguishing more appropriated clinical cases fit for this therapy, validating the dose of UCMSCs, the study definitely provides a possible future therapeutic protocol for POI and specifies certain principles of guidelines for selecting suitable patients for UCMSC transplantation.

## CONFLICT OF INTEREST

The authors declare there are no competing interests and all authors consent to publish the data.

## AUTHOR CONTRIBUTIONS

LY, YW, LL and JW performed transplant surgery and most of the experiments. WL and ZG contributed to cell isolation and culture. TL took part in designing the equipment. YF and JH are responsible for data collecting and processing. JL and HW designed the whole project, and LY, HW and F. Z. wrote the manuscript.

## Supporting information

Supplementary Material

Video S1

## Data Availability

All data generated or analysed during this study are included in this published article and its supplementary information files. In addition, the data sets used and analysed during the current study are available from the corresponding author upon reasonable request.
