# Supplementary material for: Clinical analysis of human umbilical cord mesenchymal stem cell allotransplantation in patients with premature ovarian insufficiency
Source: Cell Prolif. 2020 Oct 29;53(12):e12938. doi: 10.1111/cpr.12938 (PMC7705906; doi:10.1111/cpr.12938)
Supplement: Supplementary file 1 — Supplementary Material [file CPR-53-e12938-s001.docx]

**Supplementary information**

**Supplementary Movie 1** Display of puncture process.

Supplementary Table 1 Unilateral ovarian injection and outcomes

| **Characteristics** | **Patient 1** | | **Patient 2** | **Patient 3** |
| --- | --- | --- | --- | --- |
| **Age (years)** | 30 | | 28 | 33 |
| **Amenorrhea duration (years)** | | 10.0 | 2.0 | 9.0 |
| **BMI (kg/m^2^)** | 24.50 | | 20.80 | 24.60 |
| **Pregnancy history** | 0 | | 0 | 1 |
| **Delivery history** | 0 | | 0 | 1 |
| **E2 (pg/mL)** | 80.83 | | 23.74 | 37 |
| **LH (IU)** | 42.77 | | 11.91 | 36.95 |
| **FSH (IU)** | 94.00 | | 64.00 | 58.35 |
| **AMH (ng/mL)** | <0.06 | | <0.06 | <0.06 |
| **Ovary diameter (mm)^a, b^** | 12.0 | | 19.0 | 17.0 |
| **Ovary diameter (mm)^a, c^** | 13.0 | | 17.0 | 15.0 |
| **AFC^b^** | 0 | | 0 | 0 |
| **AFC^c^** | 0 | | 0 | 0 |
| **AFC (Total)** | 0 | | 0 | 0 |
| **Cell line** | 1 | | 1 | 1 |
| **Transplant ovary** | 1 AFC, Follow-up 1  2 AFC, Follow-up 2 | | 1 MFC, Follow-up 2  2 MFC, Follow-up 3 | 2 AFC, Follow-up 2  1 DFC, Follow-up 3  1 AFC, Follow-up 4 |
| **No transplant ovary** | No AFC | | No AFC | No AFC |

Note: BMI = body mass index; AFC = antral follicle count; DFC = dominant follicle count; MFC = mature follicle count.

^a^ Major axis; ^b^ Left ovary; ^c^ right ovary

Supplementary Table 2 MFC development after stem cell therapy

| **MFC** | **Cell line 1**  **(n = 21)** | **Cell line 2**  **(n = 40)** | ***P*** | **Total**  **(n = 61)** |
| --- | --- | --- | --- | --- |
| **Overall** | 7 (33.3%) | 12 (20.0%) | 0.789 | 19 (31.1%) |
| **First transplant** |  |  | *P_cell_* = 0.054  *P_time_* = 0.082 |  |
| Follow-up 1 | 1 (4.8%) | 5 (12.5%) | 0.609 | 6 (9.8%) |
| Follow-up 2 | 4 (20.0%) | 6 (16.7%) | 1.000 | 10 (17.9%) |
| Follow-up 3 | 0 (0%) | 3 (30.0%) | 0.060 | 3 (10.3%) |
| Follow-up 4 | 2 (14.3%) | 2 (28.6%) | 0.844 | 4 (19.0%) |
| **Second transplant** |  |  | *P_cell_* = 0.148  *P_time_* = 0.007** |  |
| Follow-up 1 | 0 (0%) | 3 (8.6%) | 0.545 | 3 (6.0%) |
| Follow-up 2 | 0 (0%) | 1 (4.5%) | 1.000 | 1 (2.8%) |
| Follow-up 3 | 1 (8.3%) | 0 (0%) | 1.000 | 1 (4.5%) |
| Follow-up 4 | 1 (12.5%) | 2 (28.6%) | 0.897 | 3 (20.0%) |
| **Third transplant** |  |  | *P_cell_* = 0.512  *P_time_* = 0.557 |  |
| Follow-up 1 | 1 (25.0%) | 1 (3.8%) | 0.253 | 2 (6.7%) |
| Follow-up 2 | 0 (0%) | 1 (4.3%) | 1.000 | 1 (3.8%) |
| Follow-up 3 | 0 (0%) | 0 (0%) |  | 0 (0%) |
| Follow-up 4 | 0 (0%) | 0 (0%) |  | 0 (0%) |

Note: Quantitative variables with heterogeneous variance were expressed as median (1st quartiles, 3rd quartiles); P_time_ and P_cell_ were the statistical significance among different time points and different cell lines.

**P* < 0.05; ** *P* < 0.01; *** *P* < 0.001.

Supplementary Table 3 IVF cycles and outcomes

| **Case no.** | **Oocyte retrieval** | **Oocyte** | **Embryo transplantation** | **Pregnancy** |
| --- | --- | --- | --- | --- |
| **1** | 3 | 1 | 1 | 1 |
| **2** | 1 |  |  |  |
| **3** | 2 | 2 |  |  |
| **4** | 1 | 1 |  |  |
| **5** | 1 | 1 | 1 |  |
| **6** | 2 | 1 | 1 |  |
| **7** | 3 | 2 |  |  |
| **8** | 2 | 1 | 1 |  |
| **9** | 3 |  |  |  |
| **10** | 1 | 1 | 1 |  |
| **11** | 4 | 1 | 1 | 1 |
| **12** | 5 | 2 |  |  |
| **13** | 2 | 2 | 4 |  |
| **14** | 7 | 2 | 2 | 1 |
| **15** | 2 | 1 |  |  |

Supplementary Table 4 Pregnancy and neonatal outcomes

| **Case no.** | 1 | 2 | 3 | 4 |
| --- | --- | --- | --- | --- |
| **Type of pregnancy** | ICSI | ICSI | Natural conception | ICSI |
| **Type of delivery** | cesarean section | vaginal delivery | cesarean section | vaginal delivery |
| **Gestational weeks** | 36 | 36 | 40 | 39+5 |
| **Pregnancy outcomes** | live birth | live birth | live birth | live birth |
| **Sex of infants** | female | male | female | female |
| **Birth height (cm)** | 48 | 46 | 50 | 48 |
| **Birth weight (g)** | 2400 | 2500 | 3725 | 2680 |
| **Birth defects** | no | no | no | no |
| **Pregnancy complications** | placenta previa | PROM | no | hypertension |
| **Height at 1 year (cm)** | 78 | NA | 75 | NA |
| **weight at 1 year(g)** | 8.9 | NA | 8.5 | NA |
| **Birth defects at 1 year** | no | no | no | no |

Note: ICSI = intracytoplasmic sperm injection; PROM = Premature rupture of membranes; NA = not applicable.

Supplementary Table 5 Characteristics of patients with AFC preoperatively

| **Characteristics** | **AFC**  **(n = 19)** | **Non AFC**  **(n = 42)** | ***P*** |
| --- | --- | --- | --- |
| **Age (years)** | 31 (29, 33) | 31 (28, 34) | 0.650 |
| **Amenorrhea duration (years)** | 2 (0.33, 3) | 4 (2, 9) | 0.001** |
| **BMI (kg/m^2^)** | 22.6 (20, 24.4) | 21.05 (19.75, 24.35) | 0.279 |
| **Pregnancy history** | 0 (0, 1) | 0 (0, 0.25) | 0.517 |
| **Delivery history** | 0 (0, 0) | 0 (0, 0) | 0.355 |
| **E2 (pg/mL)** | 79 (37, 128) | 37 (37, 77.21) | 0.083 |
| **LH (IU)** | 26.67 (19.52, 31.89) | 24.06 (18.97, 34.53) | 0.809 |
| **FSH (IU)** | 56.49 (40.45, 62.8) | 52.96 (44.73, 66.37) | 0.436 |
| **AMH (ng/mL)** |  |  | 0.114 |
| **< 0.06** | 11 (57.9%) | 34 (81.0%) |  |
| **≥ 0.06** | 8 (42.1%) | 8 (19.0%) |  |
| **Ovary diameter (mm)^a, b^** | 21 (18,24) | 17 (14,19) | 0.001** |
| **Ovary diameter (mm)^a, c^** | 21 (18, 25) | 16 (15,19) | <0.001*** |

Note: Nonnormally distributed continuous variables were expressed as median (1st quartiles, 3rd quartiles) and statistical differences were calculated using Mann-Whitney test or Kruskal-Wills test; categorical variables were expressed as N (%) and statistical differences were calculated using χ2 or Fisher's Exact Test.

BMI = body mass index; AFC = antral follicle count.

^a^ Mean diameter; ^b^ Left ovary; ^c^ right ovary

* *P* < 0.05; ** *P* < 0.01; *** *P* < 0.001.

Supplementary Table 6 Association between age and therapeutic effect

| **Age (years)** | **< 30**  **(n = 21)** | **30-35**  **(n = 31)** | **≥ 35**  **(n = 9)** | ***P*** |
| --- | --- | --- | --- | --- |
| **First transplant** |  |  |  |  |
| AFC | 11 (52.4%) | 18 (58.1%) | 5 (55.6%) | 0.921 |
| DFC | 5 (23.8%) | 7 (22.6%) | 1 (11.1%) | 0.832 |
| MFC | 7 (33.3%) | 11 (35.5%) | 1 (11.1%) | 0.367 |
| AFC & DFC & MFC | 13 (61.9%) | 22 (71.0%) | 6 (66.7%) | 0.791 |
| **Second transplant** |  |  |  |  |
| AFC | 4 (23.5%) | 11 (44.0%) | 6 (66.7%) | 0.096 |
| DFC | 2 (11.8%) | 2 (8.0%) | 1 (11.1%) | 1.000 |
| MFC | 2 (11.8%) | 3 (12.0%) | 1 (11.1%) | 1.000 |
| AFC & DFC & MFC | 4 (23.5%) | 12 (48.0%) | 6 (66.7%) | 0.085 |
| **Third transplant** |  |  |  |  |
| AFC | 1 (9.1%) | 7 (53.8%) | 5 (83.3%) | 0.007**^a^ |
| DFC | 0 (0%) | 1 (7.7%) | 0 (0%) | 1.000 |
| MFC | 2 (18.2%) | 2 (15.4%) | 0 (0%) | 0.812 |
| AFC & DFC & MFC | 3 (27.3%) | 7 (53.8%) | 5 (83.3%) | 0.076 |
| **Total** |  |  |  |  |
| AFC | 12 (57.1%) | 22 (71.0%) | 8 (88.9%) | 0.213 |
| DFC | 6 (28.6%) | 9 (29.0%) | 1 (11.1%) | 0.536 |
| MFC | 7 (33.3%) | 11 (35.5%) | 1 (11.1%) | 0.367 |
| AFC & DFC & MFC | 14 (66.7%) | 23 (74.2%) | 8 (88.9%) | 0.446 |

Note: categorical variables were expressed as N (%). Statistical differences were calculated using χ2 or Fisher's Exact Test. AFC = antral follicle count; DFC = dominant follicle count; MFC = mature follicle count; Total = Follicle outcomes of all three transplants.

^a^ Significantly different from Group <30 and Group≥35.

* *P* < 0.05; ** *P* < 0.01; *** *P* < 0.001.

Supplementary Table 7 Association between BMI and therapeutic effect

| **BMI (kg/m^2^)** | **Total**  **(n = 61)** | **< 25**  **(n = 50)** | **≥ 25**  **(n = 11)** | ***P*** |
| --- | --- | --- | --- | --- |
| **First transplant** |  |  |  |  |
| AFC | 34 (55.7%) | 30 (60.0%) | 4 (36.4%) | 0.190 |
| DFC | 13 (21.3%) | 12 (24.0%) | 1 (9.1%) | 0.429 |
| MFC | 19 (31.1%) | 16 (32.0%) | 3 (27.3%) | 1.000 |
| AFC & DFC & MFC | 41 (67.2%) | 35 (70.0%) | 6 (54.5%) | 0.526 |
| **Second transplant** |  |  |  |  |
| AFC | 21 (41.2%) | 20 (46.5%) | 1 (12.5%) | 0.119 |
| DFC | 5 (9.8%) | 5 (11.6%) | 0 (0%) | 0.580 |
| MFC | 6 (11.8%) | 6 (14.0%) | 0 (0%) | 0.572 |
| AFC & DFC & MFC | 22 (43.1%) | 21 (48.8%) | 1 (12.5%) | 0.129 |
| **Third transplant** |  |  |  |  |
| AFC | 13 (43.3%) | 10 (45.5%) | 3 (37.5%) | 1.000 |
| DFC | 1 (3.3%) | 1 (4.5%) | 0 (0%) | 1.000 |
| MFC | 4 (13.3%) | 4 (18.2%) | 0 (0%) | 0.550 |
| AFC & DFC & MFC | 15 (50.0%) | 12 (54.5%) | 3 (37.5%) | 0.680 |
| **Total** |  |  |  |  |
| AFC | 42 (68.9%) | 36 (72.0%) | 6 (54.5%) | 0.294 |
| DFC | 16 (26.2%) | 15 (30.0%) | 1 (9.1%) | 0.259 |
| MFC | 19 (31.1%) | 16 (32.0%) | 3 (27.3%) | 1.000 |
| AFC & DFC & MFC | 45 (73.8%) | 38 (76.0%) | 7 (63.6%) | 0.642 |

Note: categorical variables were expressed as N (%). Statistical differences were calculated using χ2 or Fisher's Exact Test. AFC = antral follicle count; DFC = dominant follicle count; MFC = mature follicle count; Total = Follicle outcomes of all three transplants.
